# Supplementary material for: Clinical characteristics and predictive models for hospitalized patients with COVID-19 combined with bacterial pneumonia
Source: PLoS One. 2025 Nov 21;20(11):e0336668. doi: 10.1371/journal.pone.0336668 (PMC12637964; doi:10.1371/journal.pone.0336668)
Supplement: Attachment — Submitted filename: PLOS REVIEW.docx [file pone.0336668.s001.docx]

The idea of the manuscript is good, although the COVID-19 as the main topic has switched into common disease.

The manuscript meet the requirement that describe a technically sound piece of scientific research with data that supports the conclusions. The conclusions is drawn appropriately based on the data presented.

The statistical analysis been performed appropriately within this study.

In general, the ideas may still apply to other lower respiratory tract infection, therefore some outstanding issues need to be clarified properly as bellow:

1. The title is clear and well-described the purpose of the study outcome among the population.

2. In the Introduction: the rationale observing the clinical characteristics is clear enough, however since the COVID-19 has moved to endemic state, similar with seasonal influenza, it needs more elaborate argumentation with present situation. Please elaborate with other situation which mimic with COVID-19 with bacteria infection, that common occur in healthcare facilities.

3. May the predictive model of the study applied with other viral bacterial infection, e.g. bacterial + viral sepsis ? If yes, please add information into the manuscript, especially into the introduction.

4. In the method section:

- The immune response may be altered due to comorbidities in the population of COVID-19, however, there is no information about the screening of comorbidities among the population, as the enrollment criteria.

- The population was taken between Dec 2022 — Feb 2023, How does the impact of the virus variant to co-infection/secondary bacterial infection? As we know that the shifting of variant play role during the pandemic, which implies to the clinical characteristics and disease outcome.

5. Result section 3.2: The paragraph written is difficult to follow, author need simplify and highlight the important finding of the study.

6. The Figure 2 describes the stratification of patient recovery and death, based on antibiotic. What is the purposes to stratified using this approach?

7. PWGNAA abbreviation seems confusing to read, may be need other abbreviation or synonym, e.g. predictive model, etc.

8. In the discussion, there is no information about the further implication of this study to the recent situation since the COVID-19 has been turned into endemic phase. As mentioned earlier, does all the predictive model applies to other URTI or severe immune response disease, e.e.g sepsis?
