# Supplementary material for: Clinical characteristics and predictive models for hospitalized patients with COVID-19 combined with bacterial pneumonia
Source: PLoS One. 2025 Nov 21;20(11):e0336668. doi: 10.1371/journal.pone.0336668 (PMC12637964; doi:10.1371/journal.pone.0336668)
Supplement: Attachment — Submitted filename: Response to Reviewers.docx [file pone.0336668.s003.docx]

**Point by Point Response to Reviewers’ Comments**

Journal Requirements:

When submitting your revision, we need you to address these additional requirements.

1. Please ensure that your manuscript meets PLOS ONE's style requirements, including those for file naming. The PLOS ONE style templates can be found at

https://journals.plos.org/plosone/s/file?id=wjVg/PLOSOne_formatting_sample_main_body.pdf and

<https://journals.plos.org/plosone/s/file?id=ba62/PLOSOne_formatting_sample_title_authors_affiliations.pdf>

**Author response**: Yes.

2. Please note that PLOS ONE has specific guidelines on code sharing for submissions in which author-generated code underpins the findings in the manuscript. In these cases, we expect all author-generated code to be made available without restrictions upon publication of the work.

Please review our guidelines at https://journals.plos.org/plosone/s/materials-and-software-sharing#loc-sharing-code and ensure that your code is shared in a way that follows best practice and facilitates reproducibility and reuse.

**Author response**: Our article does not contain any code.

3. We note that you have indicated that there are restrictions to data sharing for this study. For studies involving human research participant data or other sensitive data, we encourage authors to share de-identified or anonymized data. However, when data cannot be publicly shared for ethical reasons, we allow authors to make their data sets available upon request. For information on unacceptable data access restrictions, please see http://journals.plos.org/plosone/s/data-availability#loc-unacceptable-data-access-restrictions.

Before we proceed with your manuscript, please address the following prompts:

a) If there are ethical or legal restrictions on sharing a de-identified data set, please explain them in detail (e.g., data contain potentially identifying or sensitive patient information, data are owned by a third-party organization, etc.) and who has imposed them (e.g., a Research Ethics Committee or Institutional Review Board, etc.). Please also provide contact information for a data access committee, ethics committee, or other institutional body to which data requests may be sent.

**Author response:** The data contain potentially identifying or sensitive patient information, and the Research Ethics Committee has imposed restrictions on them. Requests for additional study-related data should be directed to the Ethics Committee of West China Hospital, Sichuan University, via email at [hxlcyjglb@163.com](mailto:hxlcyjglb@163.com).

b) If there are no restrictions, please upload the minimal anonymized data set necessary to replicate your study findings to a stable, public repository and provide us with the relevant URLs, DOIs, or accession numbers. Please see http://www.bmj.com/content/340/bmj.c181.long for guidelines on how to de-identify and prepare clinical data for publication. For a list of recommended repositories, please see https://journals.plos.org/plosone/s/recommended-repositories. You also have the option of uploading the data as Supporting Information files, but we would recommend depositing data directly to a data repository if possible.

4. In this instance it seems there may be acceptable restrictions in place that prevent the public sharing of your minimal data. However, in line with our goal of ensuring long-term data availability to all interested researchers, PLOS’ Data Policy states that authors cannot be the sole named individuals responsible for ensuring data access (http://journals.plos.org/plosone/s/data-availability#loc-acceptable-data-sharing-methods).

Data requests to a non-author institutional point of contact, such as a data access or ethics committee, helps guarantee long term stability and availability of data. Providing interested researchers with a durable point of contact ensures data will be accessible even if an author changes email addresses, institutions, or becomes unavailable to answer requests.

Before we proceed with your manuscript, please also provide non-author contact information (phone/email/hyperlink) for a data access committee, ethics committee, or other institutional body to which data requests may be sent. If no institutional body is available to respond to requests for your minimal data, please consider if there any institutional representatives who did not collaborate in the study, and are not listed as authors on the manuscript, who would be able to hold the data and respond to external requests for data access? If so, please provide their contact information (i.e., email address). Please also provide details on how you will ensure persistent or long-term data storage and availability.

**Author response:** The data contain potentially identifying or sensitive patient information, and the Research Ethics Committee has imposed restrictions on them. Requests for additional study-related data should be directed to the Ethics Committee of West China Hospital, Sichuan University, via email at [hxlcyjglb@163.com](mailto:hxlcyjglb@163.com). The datasets are available from the Ethics Committee upon reasonable request.

5. Your ethics statement should only appear in the Methods section of your manuscript. If your ethics statement is written in any section besides the Methods, please move it to the Methods section and delete it from any other section. Please ensure that your ethics statement is included in your manuscript, as the ethics statement entered into the online submission form will not be published alongside your manuscript.

**Author response:** Yes.

6. Please review your reference list to ensure that it is complete and correct. If you have cited papers that have been retracted, please include the rationale for doing so in the manuscript text, or remove these references and replace them with relevant current references. Any changes to the reference list should be mentioned in the rebuttal letter that accompanies your revised manuscript. If you need to cite a retracted article, indicate the article’s retracted status in the References list and also include a citation and full reference for the retraction notice.

**Author response:** We have reviewed our reference list to ensure that it is complete and correct.

If the reviewer comments include a recommendation to cite specific previously published works, please review and evaluate these publications to determine whether they are relevant and should be cited. There is no requirement to cite these works unless the editor has indicated otherwise.

**Author response:** The reviewer's comments do not cover the above-mentioned matters.

**Additional Editor Comments:**

**Reviewer #1**: The idea of the manuscript is good, although the COVID-19 as the main topic has switched into common disease.

Please see the upload files for detailed of comment specific related the manuscript, and author need to clarify each of the question clearly.

1. The title is clear and well-described the purpose of the study outcome among the population.

**Author response 1#:** Thank you very much for your recognition.

2. In the Introduction: the rationale observing the clinical characteristics is clear enough, however since the COVID-19 has moved to endemic state, similar with seasonal influenza, it needs more elaborate argumentation with present situation. Please elaborate with other situation which mimic with COVID-19 with bacteria infection, that common occur in healthcare facilities.

**Author response 1#****:** Thank you very much for your suggestion. According to your advice, we added that “The transition of COVID-19 has moved from a pandemic to an endemic state in recent years, akin to seasonal influenza which projects asynchronous surges of SARS-CoV-2 in different regions”in the introduction section. Besides, we added the elaborate with other situation which mimic with COVID-19 with bacteria infection, that common occur in healthcare facilities. The phenomenon of bacterial coinfections is not unique to COVID-19 and has been observed in other viral respiratory infections. Viral infections, such as those caused by influenza and respiratory syncytial virus, are well-documented to predispose individuals to bacterial infections, which can exacerbate disease severity and increase mortality rates. The revision located on Page 4, line 52-55 and line 58-62, marked red.

3. May the predictive model of the study applied with other viral bacterial infection, e.g. bacterial + viral sepsis? If yes, please add information into the manuscript, especially into the introduction.

**Author response 1#:** Thank you very much for your suggestion. We recognize that the predictive model of the study applied with other viral bacterial infection could provide more robust support for our conclusions. Consequently, we plan to incorporate a validation cohort regarding bacterial + viral sepsis in our future research.

4. In the method section:

- The immune response may be altered due to comorbidities in the population of COVID-19, however, there is no information about the screening of comorbidities among the population, as the enrollment criteria.

**Author response 1#:** Thank you very much for your suggestion. According to your advice, we added the comorbidities in the Data Retrieval section. The following data were collected: comorbidities (including hypertension, diabetes, cardiovascular disease, chronic lung/renal diseases, immune system disease, and malignancy). The revision located on Page 8, line 146-147, marked red. Table 1 shows the comorbidities. The revision located on Page 10.

- The population was taken between Dec 2022 — Feb 2023, How does the impact of the virus variant to co-infection/secondary bacterial infection? As we know that the shifting of variant play role during the pandemic, which implies to the clinical characteristics and disease outcome.

**Author response 1#:** Thank you very much for your suggestion. Our hospital tested the SARS-CoV-2 by RT-PCR test, but no subtype analysis was conducted. However, according to the Chinese Center for Disease Control and Prevention (CDC), the BA.5 subvariants was the dominant circulating variant (reached to 43%) at the time (around December 2022 to March 2023) when conducting this study in Chengdu, China. We added above information in the Enrollment criteria. The revision located on Page 6, line 108-111, marked red.

5. Result section 3.2: The paragraph written is difficult to follow, author need simplify and highlight the important finding of the study.

**Author response 1#:** Thank you very much for your suggestion. We modified it, and simplify and highlight the important finding of the study. Out of 1060 positive culture samples (Fig. 1a), the predominant isolates were Acinetobacter baumanii (244 cases, 23.02%), Klebsiella pneumoniae (204 cases, 19.25%), and Pseudomonas aeruginosa (90 cases, 8.49%). Among these, 833 sputum samples were positive for bacteria (Fig. 1b), with Acinetobacter baumanii (210 cases, 25.21%), Klebsiella pneumoniae (165 cases, 19.81%), and Pseudomonas aeruginosa (83 cases, 9.96%) as the main isolates. Additionally, 168 blood cultures were positive, primarily for Acinetobacter baumanii (32 cases, 19.05%), Klebsiella pneumoniae (27 cases, 16.07%), and Enterococcus faecium (20 cases, 11.9%) (Fig. 1c). There were only 13 cases in which the results of blood culture and respiratory tract culture were consistent. The revision located on Page 12-13, line 192-211, marked red.

6. The Figure 2 describes the stratification of patient recovery and death, based on antibiotic. What is the purposes to stratified using this approach?

**Author response 1#:** Thank you very much for your suggestion. The stratification of patient recovery and death based on antibiotic use is a critical approach in managing pneumonia. The primary purpose of stratifying patients in this manner is to optimize antibiotic therapy, ensuring that patients receive the most appropriate treatment based on their specific clinical conditions and the characteristics of the pathogens involved.

7. PREDICTIVE MODEL abbreviation seems confusing to read, may be need other abbreviation or synonym, e.g. predictive model, etc.

**Author response 1#:** Thank you very much for your suggestion. We modified “PREDICTIVE MODEL abbreviation” to “predictive model”. The revision located on Page 17, line 259-260, line 263, line 265, line 267 and Table 4, marked red.

8. In the discussion, there is no information about the further implication of this study to the recent situation since the COVID-19 has been turned into endemic phase. As mentioned earlier, does all the predictive model applies to other URTI or severe immune response disease, e.e.g sepsis?

**Author response 1#:** Thank you very much for your suggestion. Despite the transition of the COVID-19 pandemic to an endemic phase, the virus is expected to persist for an extended period. The occurrence of COVID-19 complicated by bacterial pneumonia will continue to be a prevalent clinical scenario. The findings of this study hold significant clinical relevance for the early diagnosis of COVID-19 complicated by pneumonia and for reducing mortality in these patients. The revision located on Page 24, line 391-395, marked red.

However, this study did not include research on patients with upper respiratory tract infections (URTI), sepsis, or similar conditions. Future research will focus on these populations to further validate the predictive model and assess its applicability to infections in other parts. We have added the above limitations in the Discussion section. The revision located on Page 24-25, line 402-405, marked red.

**Reviewer #2**: The study is devoted to an essential problem – finding a way to predict the likelihood of bacterial pneumonia in patients with COVID-19. It is known that there are no reliable diagnostic criteria, which leads to hypo- and hyperdiagnosis and, accordingly, unjustified prescription of antibiotics.
However, despite the relevance of the problem raised by the paper, there are significant questions on methodology and the results that I would ask to clarify.

Materials and Methods
1. Selection of the population to be analysed
Why did the authors choose to combine bacterial pneumonia as coinfections and secondary infection (namely nosocomial pneumonia) into one group? These are different cohorts of patients, with different risk factors on the development of pneumonia, different aetiology, prognosis, and decisions to be made. It is well known that if we select good studies, bacterial coinfections in COVID-19 remain rare, but secondary superinfections, including nosocomial pneumonia, are frequent and indeed significantly worsen the prognosis.

**Author response 2#:** Thank you very much for your suggestion. We carefully re-examined the data and found no co-infections. A total of 1,386 cases involved combined bacterial pneumonia, all of which were classified as secondary bacterial pneumonia. The revision located on Page 9, line 174-175, marked red.

2. Inclusion/exclusion criteria
- One of the exclusion criteria requires clarification:

3) positive sputum culture results considered to reflect colonisation or contamination on the basis of clinical judgement. What does clinical judgement mean in this case to assess the results as insignificant? What specific criteria were used?
- Selection of the control group: why the absence of sputum (it is also quite common in bacterial pneumonia, especially at the onset of the disease) as well as recovery without AB therapy allowed to exclude bacterial co-/superinfection?

**Author response 2#:** Thank you very much for your suggestion. Two researchers, each holding the title of Associate Chief Physician or higher, independently assessed whether cases constituted contamination, colonization, or infection, adhering to the National Health Commission of the People's Republic of China (2001) Diagnostic Criteria for Nosocomial Infection (Trial). In instances of disagreement, a face-to-face discussion was conducted to reach a consensus. If a case was identified as contamination or colonization, the treatment plan remained unchanged, although relevant indicators and clinical symptoms were closely monitored. The revision located on Page 7, line 129-135, marked red. References: The National Health Commission of the People's Republic of China (2001) Diagnostic Criteria for Nosocomial Infection (Trial). Chinese Medical Journal, 81, 314-320. DOI. 10.3760/j:issn:0376-2491.2001.05.027

Patients with absence of sputum or negative sputum cultures and without any radiological evidence of bacterial pneumonia were selected as the control group. The revision located on Page 8, line 139-140, marked red.

3. When choosing bacterial pneumonia, you have relied only on sputum culture, which allow the detection of only typical bacterial pathogens. What was the case with the detection of routinely non-culturable but significant community-acquired bacteria such as M. pneumoniae, L. pneumophila?

**Author response 2#:** Thank you very much for your suggestion. Regarding the detection of pathogens in sputum, in addition to conventional sputum culture, some patients underwent additional pathogen detection methods, including respiratory virus and pathogenic bacterial nucleic acid testing, *Legionella* antigen testing, *Mycoplasma pneumoniae* antibody testing, *Chlamydia pneumoniae* antibody testing, and next-generation sequencing (NGS). The revision located on Page 7, line 115-119, marked red.

4. You developed the pneumonia model, why did you evaluate the urine culture data?
Results

**Author response 2#:** Thank you very much for your suggestion. Urine information was excluded from the analysis.

1. Why, out of 5,358 COVID-19 patients, you could only include 1,386 in the study group and 408 in the control group. Explain the reasons.

**Author response 2#:** Thank you very much for your suggestion. Cases were screened according to predefined inclusion and exclusion criteria, and those that could not be definitively diagnosed were omitted.

2. Please explain what was the proportion of bacterial co-infection and secondary/ superinfection in the study group? This is important in terms of understanding the structure of the pathogens that are presented in the article.

**Author response 2#:** Thank you very much for your suggestion. We carefully re-examined the data and found no co-infections. A total of 1,386 cases involved combined bacterial pneumonia, all of which were classified as secondary bacterial pneumonia. The revision located on Page 9, line 174-175, marked red.

3. When assessing the results of sputum culture, was the quality of samples evaluated, in particular, were the results of Gram staining taken into account?

**Author response 2#:** Thank you very much for your suggestion. We evaluated the sputum specimens by considering their quality, the results of sputum smears, clinical manifestations, and imaging examinations, ultimately finding that the comprehensive assessment was consistent with bacterial pneumonia.

4. How were cases of bacteriemia interpreted? Were the isolated microorganisms considered to be pathogens of pneumonia? Did the blood cultures and respiratory sample cultures match?

**Author response 2#:** Thank you very much for your suggestion. Bacteremia was interpreted based on the presence of symptoms indicative of bloodstream infection, alongside positive blood culture results. It seems that these cannot be directly attributed to the causative bacteria of pneumonia. There were only 13 cases in which the results of blood culture and respiratory tract culture were consistent. The revision located on Page 13, line 210-211, marked red.

5. Why does the article provide data on AB prescription if the quality of their usage is not further analyzed? The prescriptions are given only for AB groups, including beta-lactams, that contain drugs with a fundamentally different spectrum of activity.

**Author response 2#:** Thank you very much for your suggestion. We conducted a more detailed analysis of antibiotic prescriptions by examining the specific types of drugs utilized within each major antibiotic category. The revision located on Page 14-17, Table 2, marked red.

6. The choice of cut-off points for WBCs and PCT level needs to be explained.
Discussion

**Author response** **2#:** Thank you very much for your suggestion. The model was developed utilizing the actual values of WBCs and PCT, yet its predictive efficacy was inferior to that of the model employing the cut-off values for WBCs and PCT. The selection of the cut-off values for WBCs and PCT levels was made to make the model more convenient to use, especially in areas with scarce medical resources. The revision located on Page 23, line 367-369, marked red.

1. Please explain what treatment patients received for COVID-19. You claim that
SARS-CoV-2 was a major risk factor for bacterial co-/superinfections. However, treatment, particularly the use of glucocorticosteroids and immunosuppressants, as well as the presence of various invasive devices in severe cases, are equally important risk factors for bacterial superinfections.

**Author response** **2#:** Thank you very much for your suggestion. We have incorporated the treatment modalities—glucocorticosteroids, immunosuppressants, invasive mechanical ventilation, and hemodialysis—administered to COVID-19 patients. To streamline the indicators, we have included variables such as gender, age, and laboratory indicators in the model. However, we have not incorporated specific treatment measures. The revision located on Page 10, Table 1 and Page 17, line 248-249, marked red.

2. It would be useful to add to the discussion at what stage you propose to use the developed model for predicting bacterial pneumonia – upon admission, after 48 hours, or at other times?

**Author response 2#:** Thank you very much for your suggestion. It can be used in multiple time periods, especially when there is suspicion of secondary infection. The revision located on Page 24, line 387-388, marked red.

3. The section on research limitations must be expanded; in particular, it does not mention that bacterial pathogens were identified solely on the basis of sputum culture.
Conclusion

**Author response 2#:** Thank you very much for your suggestion. Bacterial pathogens were identified through a comprehensive analysis involving sputum culture, nucleic acid testing for respiratory viruses and pathogenic bacteria, Legionella antigen testing, Mycoplasma pneumoniae antibody testing, Chlamydia pneumoniae antibody testing, and next-generation sequencing (NGS). The revision located on Page 7, line 115-119, marked red.

You conclude that bacterial infection in COVID-19 has an adverse effect on prognosis. Was the risk of mortality adjusted in the study and control groups for confounding factors such as age and comorbidities, which are themselves associated with a poorer prognosis?

**Author response 2#:** Thank you very much for your suggestion.To streamline the indicators, we have included variables such as gender, age, and laboratory indicators in the model. However, we have not incorporated specific comorbidities. The revision located on Page 17, line 248-249, marked red.
